# Muscone improves hypoxia/reoxygenation (H/R)-induced neuronal injury by blocking HMGB1/TLR4/NF-κB pathway *via* modulating microRNA-142

**DOI:** 10.7717/peerj.13523

**Published:** 2022-07-15

**Authors:** Weihua Ren, Fucheng Zhao, Yanru Han, Zhenzhou Liu, Jianli Zhai, Kui Jia

**Affiliations:** Department of Integrated Chinese and Western Medicine, The First Affiliated Hospital of Xinxiang Medical University, Weihui, Henan, China

**Keywords:** Muscone, MicroRNA-142-5p, HMGB1/TLR4/NF-κB pathway, Cerebral hypoxia injury

## Abstract

Previous reports have indicated that natural muscone has neuroprotective effects against cerebral hypoxia injury; however, little is known in regards to its pharmacological mechanism. In this study, we tried to evaluate the neuroprotective effects and mechanisms of muscone against cerebral hypoxia injury using an *in vitro* model. The cerebral hypoxia injury cell model was produced by hypoxia/reoxygenation (H/R). The cell viability and apoptosis were measured using the cell counting Kit-8 and the Annexin V-FITC/PI Apoptosis Detection kit, respectively. To screen microRNAs regulated by muscone, we analyzed the gene expression datasets of GSE84216 retrieved from gene expression omnibus (GEO). Here, it was demonstrated that muscone treatment significantly alleviated the cell apoptosis, oxidative stress and inflammation in H/R-exposed neurons. Subsequently, through analyzing GSE84216 from the GEO database, miR-142-5p was markedly upregulated by treatment of muscone in this cell model of cerebral hypoxia injury. Further experiments revealed that downregulation of miR-142-5p eliminated the neuroprotective effects of muscone against H/R induced neuronal injury. Additionally, high mobility group box 1 (HMGB1), an important inflammatory factor, was identified as a direct target of miR-142-5p in neurons. Meanwhile, we further demonstrated that muscone could reduce the expression of HMGB1 by upregulating miR-142-5p expression, which subsequently resulted in the inactivation of TLR4/NF-κB pathway, finally leading to the improvement of cell injury in H/R-exposed neurons. Overall, we demonstrate for the first time that muscone treatment alleviates cerebral hypoxia injury in *in vitro* experiments through blocking activation of the TLR4/NF-κB signaling pathway by targeting HMGB1, suggesting that muscone may serve as a potential therapeutic drug for treating cerebral hypoxia injury.

## Introduction

Brain hypoxia is one of the most common phenomenon that results in impaired circulation and metabolic disruptions, which can trigger tissue infarctions, mainly in the brain because it consumes oxygen at a high rate (Miyamoto & Auer, 2000). Hypoxia is also a major cause of foetal brain damage with long-lasting behavioral implications, including mental retardation and cognitive deficit (Golan & Huleihel, 2006; Nyakas, Buwalda & Luiten, 1996). This condition is mainly associated with oxygen free radicals, inflammation and oxidative stress, which contributes to neurodegeneration and apoptosis (Wu et al., 2017; Zhou et al., 2016; Zhou et al., 2015). Therefore, inhibition of the oxidative stress, cell apoptosis and inflammatory response is one of the most important approaches for protection against hypoxia induced damage, such as the use of agents with anti-oxidant, anti-apoptosis and anti-inflammatory effects.

Muscone, 3-methylcyclopentadecanone, is the main ingredient of traditional Chinese medicine musk (Du et al., 2018; Wang et al., 2014). In recent years, numbers of studies have demonstrated that muscone exhibits the neuroprotective effects, and the protective mechanisms may be attributed to its anti-oxidant, anti-inflammatory and anti-apoptotic properties. For example, Wei et al. (2012) demonstrated that muscone significantly improved middle cerebral artery occluded (MCAO) induced ischemic cerebral dysfunctions in rats. Zhou et al. (2020) found that muscone could attenuate neuroinflammation and neuronal damage in a rat model of cervical spondylotic myelopathy. Liu et al. (2020) showed that muscone treatment displayed neuroprotective effects in mice model of Alzheimer’s disease (AD). Furthermore, a previous evidence suggests that muscone exerted cerebral protective effect on traumatic brain injury model rats (Jiang et al., 2016). However, the molecular mechanism involved the neuroprotective effects of muscone remain to be elucidated.

MicroRNAs (miRNAs) are ∼22-nucleotide single-stranded noncoding RNA, which modulate post-transcriptional regulation of target genes by binding the complementary sequences in the 3′-UTR of the target mRNA (Bartel, 2004). Increasing studies have indicated the involvement of miRNAs in hypoxia induced injury in different organs (Ghosh et al., 2010; Wang et al., 2010). For example, overexpression of miR-210 was demonstrated to protect PC-12 cells against hypoxia-induced injury by targeting Bcl-2 adenovirus E1B 19 kDa-interacting protein 3 (BNIP3), involving the PI3K/AKT/mTOR signal pathway (Luan et al., 2017). Overexpression of miR-494 upregulated HIF-1α expression through activating PI3K/Akt pathway under both normoxia and hypoxia, and had protective effects against hypoxia-induced apoptosis in L02 cells (Sun et al., 2013). Previous studies have demonstrated that some natural agents exert their biological functions against cerebral hypoxia injury through regulating miRNAs. For instance, Theaflavin was demonstrated to improve cerebral injury through regulating miR-128-3p in rats (Li et al., 2019). Wang et al. (2020) found that Safflor Yellow B (SYB) could attenuate brain injury *via* inhibition of miR-134 expression in rats. Thus, it is not surprising that muscone may alter the expression profile of miRNA to modulate cell apoptosis, inflammatory response and oxidative stress to improve cerebral hypoxia injury.

In the present study, we used an H/R-induced HT22 cell injury model to examine the potential therapeutic effects of muscone on cerebra hypoxia injury, and investigate its potential molecular mechanism. This is the first report about the functions of muscone in cerebral hypoxia injury in neurons. Our findings provide a potential new way for the therapy of cerebral hypoxia injury.

## Materials and Methods

### Cell culture and drug

The mouse hippocampal neuron line HT22 was purchased from the Cell Culture Center of the Shanghai Institute (Shanghai, China) and cultured in DMEM (Gibco, Thermo Fisher Scientific, Waltham, MA, USA) supplemented with 10% fetal bovine serum (FBS) and 1% penicillin and streptomycin (Sigma-Aldrich, St. Louis, MO, USA) in a humidified incubator with 5% CO_2_ at 37 °C. The medium was changed every 2 to 3 days, and the cells were passaged two times/week. The cells between passages five and 10 were subjected to H/R model. Muscone (cat no. sc-200528A) was obtained from Santa Cruz Biotechnology, Santa Cruz, CA, USA and was dissolved in DMSO (St. Louis, MO, United States) at 10 mm, and stored at −80 °C.

### Induction of hypoxia/reoxygenation (H/R) model

The HT22 cells with entire media were plated 12 h before the experiment, and at the beginning of the experiment, the HT22 cells cultured without glucose (glutamate) and FBS were transferred to an anoxia environMent (92% N_2_, 3% O_2_ and 5% CO_2_) at 37 °C for 8 h. Subsequently, culture media was replaced with DMEM medium containing 4.5 g/l glucose and supplemented with 10% FBS (Invitrogen, Thermo Fisher Scientific, Inc., Waltham, MA, USA), 100 U/ml penicillin and 100 U/ml streptomycin (Solarbio Science & Technology Co., Ltd., Beijing, China) and the cells were cultured under normoxic conditions (95% air and 5% CO_2_) for 48 at 37 °C to induce reoxygenation. After reoxygenation, cells were harvested for analyses.

In addition, the cells were divided into four groups including control group, H/R group, H/R + DMSO group and H/R + Muscone group. Also, the H/R + Muscone group included two subgroups, to which two different concentrations of Muscone (100 nM and 300 nM) were added 1 h prior to the initiation of hypoxia. Cells in the H/R, H/R + DMSO and H/R + Muscone groups were incubated for 8 h in a hypoxia condition and were then subjected to 48 of reoxygenation under normoxic conditions.

### Experimental protocols

To investigate the role of miR-142-5p in the protection of Muscone against H/R injury, HT22 cells were divided into a control group, H/R group, H/R + Muscone group, H/R + Muscone + inhibitor negative control (NC) group, H/R + Muscone + miR-142-5p inhibitor group. In H/R + Muscone + miR-142-5p inhibitor/inhibitor NC groups, HT22 cells were transfected with 50 nM miR-142-5p inhibitor/inhibitor NC, followed by treatment with Muscone for 48 h immediately after 1 h the initiation of hypoxia.

### Cell transfection

miR-142-5p mimics, miR-142-5p inhibitor or miR NC, were synthesized by GenePharma (Shanghai, China). miR-142-5p mimics/inhibitor at a final concentration of 50 nM was transfected into HT22 cells using Lipofectamine® 3,000 (Invitrogen, Thermo Fisher Scientific, Inc., Waltham, MA, USA), according to the manufacturer’s protocols. After 24 h following transfection, HT22 cells were treated with muscone before 1 h exposure to anoxia environMent, then cells were employed for further analysis. The efficiency of transfection was evaluated by RT-qPCR.

### Cell viability

For the detection of cell viability, HT22 cells (5 × 10^3^/well) were seeded in 96-well plates and incubated in corresponding medium supplemented with 10% FBS for 24 h. Then the cells were handled as describe above, the cell viability was measured using Cell counting Kit-8 (CCK-8; Dojindo, Kumamoto, Japan) assay. A total of 10 µl cell counting Kit-8 solution (Dojindo, Kumamoto, Japan) was added into each well and incubated at 37 °C for a further 2 h, the absorbance was read at 450 nM using a microplate reader (Model 680; Bio-Rad Laboratories, Inc., Hercules, CA, USA).

### Cell apoptosis assay

Cell apoptosis was evaluated using an Annexin V-FITC/PI Apoptosis Detection kit (BD Biosciences, Mountain View, CA, United States) according to the manufacturer’s instructions. Briefly, the treated cells were and dissociated with 0.25% trypsin, after which the cells were collected by centrifugation (2,000×*g*, 5 min) and washed twice by ice-cold PBS. Subsequently, the cells at 1 × 10^6^ were resuspended in 200 μl binding buffer with 10 μl Annexin V-fluorescein isothiocyanate and 5 μl propidium iodide and incubated in the dark for 30 min, and the number of cells was determined using flow cytometry (BD FACSCalibur; BD Biosciences, San Jose, CA, United States) and analyzed by FlowJo 8.7.1 software (Ashland, OR, USA). The lower left quadrant (Q4) was normal negative cells (FITC−/PI−). The lower right quadrant (Q3) was the early apoptotic cells (FITC+/PI−). The upper right quadrant (Q2) was the late apoptotic or necrotic cells (FITC+/PI+). The upper left quadrant (Q1) represented the mechanically damaged cells (FITC−/PI+). Apoptotic rate = ((early apoptotic cells + late apoptotic cells)/total number of cells) × 100%.

### The caspase 3 activity assay

Caspase-3 activity was measured using a Caspase-3 Activity kit (Beyotime Institute of Biotechnology, Jiangsu, China) according to the manufacturer’s protocol. The optical density was then detected at 405 nM using a microplate reader (Model 680; Bio-Rad Laboratories, Inc., Hercules, CA, USA).

### Measurement of intracellular ROS level

For ROS measurements, a Reactive Oxygen Species Assay Kit (Beyotime Biotechnology, Haimen, Jiangsu, China) was performed. After above treatments, cells were collected and resuspended in serum-free medium that contained 2′, 7′-dichlorofluorescein-diacetate (DCFH-DA). Cells were incubated for 25 min at 37 °C, and observed using fluorescence microscopy (IX70; Olympus, Tokyo, Japan) at 200× magnification, then the ROS levels were measured at 488 nM excitation and 525 nM emission by a fluorescence spectrophotometer (BioTek, Winooski, VT, United States). Fluorescence of 10 randomly selected areas was counted with Scion Image Software (Scion Co., Frederick, MD, USA).

### Enzyme-linked immunosorbent assay (ELISA)

HT22 cells were harvested and centrifuged at 3,000×*g* for 10 min at 4 °C. Then, the supernatant was assayed for IL-1β (cat no. 96-403), IL-6 (cat no. 96-407), IFN-α (cat no. 96-416), and IL-10 (cat no. 96-408) levels in accordance with the manufacturer’s protocols. All ELISA kits were obtained from Merck-Millpore, Billerica, MA, USA.

### Measurement of SOD and MDA levels

HT22 cells were lysed using RIPA buffer (Beyotime Institute of Biotechnology, Jiangsu, China). The supernatant was collected after centrifugation at 3,000×*g* at 4 °C for 20 min. The levels of superoxide dismutase (SOD) (cat no. S0103) and malondialdehyde (MDA) (cat no. S0131) levels were detected according to the respective instructions (Jiancheng Biotechnology Co., Ltd., Nanjing, China).

### Sequencing data analysis

Profiling data GSE84216 obtained through next-generation sequencing (NGS) were downloaded from the NCBI from the Gene Expression Omnibus (GEO, http://www.ncbi.nlm.nih.gov/geo/). The original data were preprocessed using the “limma” package in R (Ritchie et al., 2015). The fold changes (FCs) in the expression of individual miRNAs were calculated, and differentially expressed miRNAs with |log2FC| > 1.0 and *p* < 0.05 were considered to be significant. Hierarchical clustering of differentially expressed miRNA was performed with dChip (version 2010.01; https://sites.google.com/site/dchipsoft/).

### Real-time quantitative PCR analysis

Total RNA was extracted from cultured cells using a mirVana™ miRNA Isolation Kit (Thermo Fisher Scientific, Waltham, MA, USA) as the manufacturer’s instructions. miRNA was reverse transcribed to cDNA using One Step PrimeScript^®^ miRNA cDNA Synthesis kit (Takara Bio, Inc., Tokyo, Japan) by incubating at 37 °C for 60 min. For detection of HMGB1 mRNA, total RNA was reverse-transcribed to cDNA using PrimeScript RT reagent kit (Takara Bio, Inc., Tokyo, Japan). Then miRNAs and mRNA expression levels were carried out using the SYBR Premix Ex Taq™ (TaKaRa, Tokyo, Japan) on the ABI PRISM 7,900 system (Thermo Fisher Scientific, Inc., Waltham, MA, USA). U6 and GAPDH were used as internal controls for miR-142-5p and HMGB1, respectively. The primers used for were as follows: miR-142-5p, RT, 5′-GTCGTATCCAGTGCAGGGTCCGAGGTATTCGCACTGGATACGACTCCATA -3′ F, 5′-CGGAGTGTAGTGTTTCCTACTT-3′; R, 5′-GCAGGGTCCGAGGTATTC-3′; U6 F, 5′-GCTTCGGCAGCACATATACTAAAAT-3′, R, 5′-CGCTTCAGAATTTGCGTGTCAT-3′; HMGB1, F, 5′-GTGCAAACTTGTCGGGAG-3′, R, 5′-CGATACTCAGAGCAGAAGAGG-3′; GAPDH F, 5′-CAGCCTCAAGATCATCAGCA-3′ and R, 5′-GTCTTCTGGGTGGCAGTGAT-3′. The relative expression of each gene was calculated using the 2^−∆∆Ct^ method (Livak & Schmittgen, 2001). The specificity of the primers for PCRs were verified by BLASTN and DNA sequencing of the obtained PCR products.

### Luciferase reporter assay

The dual-luciferase reporter vector (pmirGLO; Promega, Madison, WI, USA) harboring the wild-type and mutated HMGB1 3′-UTR were co-transfected with miR-142 mimics/inhibitor or miR-NCs into the HT22 cells using Lipofectamine 2,000 reagent (Invitrogen, Carlsbad, CA, USA). Forty-eight hour after transfection, the firefly luciferase activity was measured by dual-luciferase assays kit (Promega, Madison, WI, USA) according to the manufacturer’s instructions. Renilla luciferase activity was used as an internal control.

### NF-κB activity assay

The HT22 cells were plated in six-well tissue culture plates at a concentration of 5 × 10^4^ cells/well for 24 h. 2.5 μg of a NF-κB reporter plasmid (GenePharma, Shanghai, China) was then transfected into HT22 cells. After 6 h, the cells were washed and then transfected with 50 nM miR-142-5p inhibitor/inhibitor NC, followed by treatment with Muscone for 24 h immediately after 1 h the initiation of hypoxia. The cells were then washed in PBS and harvested in 500 μl 1X passive lysis buffer. Luciferase activity was quantified using a Promega luciferase assay kit on a luminometer. The experimental values were recorded relative to those of untreated control samples.

### Western blot

Total protein was extracted from cells using RIPA lysis buffer (Beyotime Institute of Biotechnology, Jiangsu, China) with a protease inhibitor cocktail (Sigma-Aldrich, St. Louis, MO, USA), and the protein concentration was determined by a BCA kit (Beyotime Institute of Biotechnology, Jiangsu, China). Proteins (40 µg each line) were separated on 10% SDS-PAGE gels and transferred to PVDF membranes (Millipore, Billerica, MA, USA) followed by incubation in a 5% skim milk solution for 1 h at room temperature. Subsequently, the specific primary antibodies were incubated in the membranes at 4 °C overnight, including TLR4 (1:1,000, rabbit mAb, cat. no. 14358), MyD88 (1:1,000, rabbit mAb, cat. no. 4283), nuclear-p-p65 (1:500, cat. no. 3033), p65 (1:500, rabbit mAb, cat. no. 8242), p-IκBα (1:1,000, rabbit mAb, Ser32 cat. no. 2859), IκBα (1:1,000, rabbit mAb, cat. no. 4812), HMGB1 (1:500, rabbit mAb, cat no. 6893) and β-actin (1:2,000, rabbit mAb, cat. no. 4970). Subsequently, the corresponding goat anti-rabbit secondary antibodies (cat. no. 7074, 1:2,000) were added into the membranes for 1 h at room temperature. All antibodies were obtained from Cell Signaling Technology, Inc., Danvers, MA, USA. The results were visualized with a chemiluminescence detection system (Millipore, Billerica, MA, USA) and the quantification of the bands was performed using Quantity One software (Bio-Rad, Hercules, CA, USA). Protein levels in cells were presented as fold change normalized to an endogenous reference (β-actin protein).

### Statistical analysis

All statistical data were analyzed using GraphPad Prism 5.0 software (GraphPad Software, Inc., San Diego, CA, USA). Data are expressed as the mean ± SD. Statistical analysis was performed by unpaired Student’s t-test or one-way ANOVA, followed by *post hoc* test. Pearson’s method was used in correlation test. *p* < 0.05 was considered to indicate a statistically significant difference.

## Results

### Muscone improved H/R-induced HT22 cell injury

**Figure 1:**
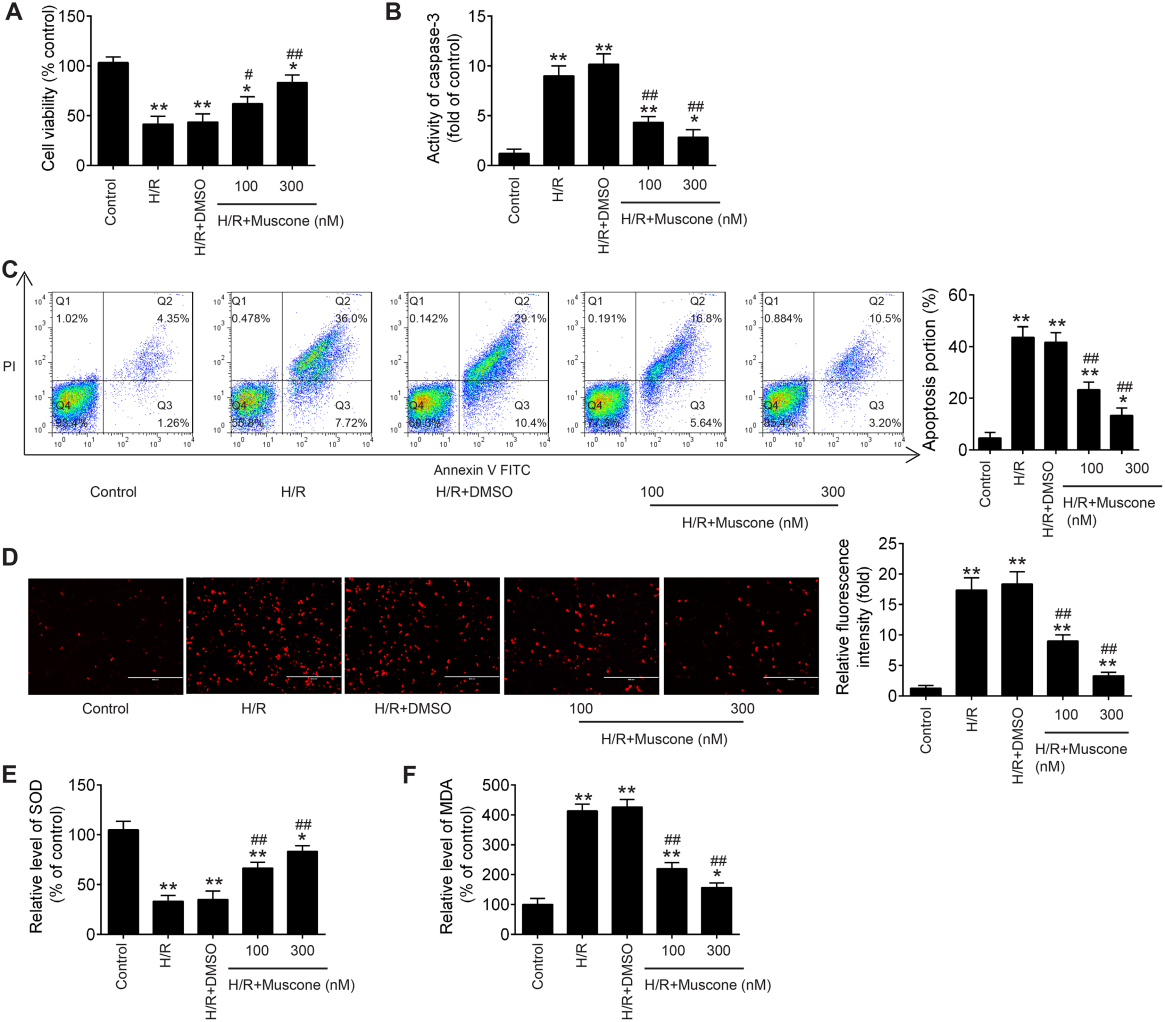
Muscone alleviated H/R-induced inflammation, oxidative stress and apoptosis in HT22 cells. HT22 cells were treated with muscone (100 nm and 300 nm) before H/R exposure. Then, cells were harvested for subsequent experiments. (A) The cell viability of each group was determined by CCK‑8 assay. (B) The activity of caspase was measured by Caspase 3 Activity kit. (C) The rate of apoptosis was detected by Annexin V/PI double staining followed by flow cytometry. (D) ROS production was detected by DCFH-DA assay. (E, F) The levels of SOD and MDA were assessed using commercial kits. Data are presented as the mean ± SD of three independent experiments. *p < 0.05 and **p < 0.01 *vs*. control group; #*p* < 0.05, ##*p* < 0.01 *vs*. H/R group.

In the current study, to explore whether Muscone could alleviate H/R-induced HT22 neurons injury, hypoxia/reoxygenation (H/R)-induced neurons injury model was established through 8 h of hypoxia and 48 h of reoxygenation in HT22 neurons. As shown in Fig. 1A, CCK-8 assay showed that H/R treatment can obviously decline the cell viability compared with the control group, and this influence was attenuated by Muscone (F = 39.1, df = 4, *p* < 0.01). Next, we detected the regulatory effect of Muscone (100 nM and 300 nM) on H/R-induced neuronal apoptosis. It was found that the activity of caspase 3 was dramatically increased in H/R group compared with the control group, whereas Muscone significantly decreased the activity of caspase 3 in HT22 neurons following H/R treatment (Fig. 1B; F = 71.1, df = 4, *p* < 0.01). Additionally, flow cytometry revealed that, compared with the control group, H/R increased the apoptotic rate of HT22 neurons. However, Muscone treatment significantly attenuated the cell apoptosis ratio caused by H/R (Fig. 1C; F = 85.5, df = 4, *p* < 0.01). These results suggested that Muscone reduced H/R-induced apoptosis rate in HT22 neurons. Furthermore, the indication of intracellular oxidative stress, the ROS and MDA levels were much higher in the H/R group than that in the control group. Of note, there was significant reduction in ROS and MDA level in the Muscone-treated group compared with the H/R group (Figs. 1D and 1E; F = 89.89, df = 4, *p* < 0.01; F = 57.62, df = 4, *p* < 0.01). In parallel with the increase in ROS and MDA, we also observed that the activity of SOD was significantly decreased in H/R group and significantly restored by Muscone treatment (Fig. 1F; F = 151.9, df = 4, *p* < 0.01). Moreover, these effects of Muscone were in a dose-dependent manner, especially in 300 nM group. Collectively, these findings indicate that Muscone could improve H/R-induced HT22 neurons injury by reducing cell apoptosis and oxidative stress.

### Muscone suppressed H/R-induced inflammatory response

**Figure 2:**
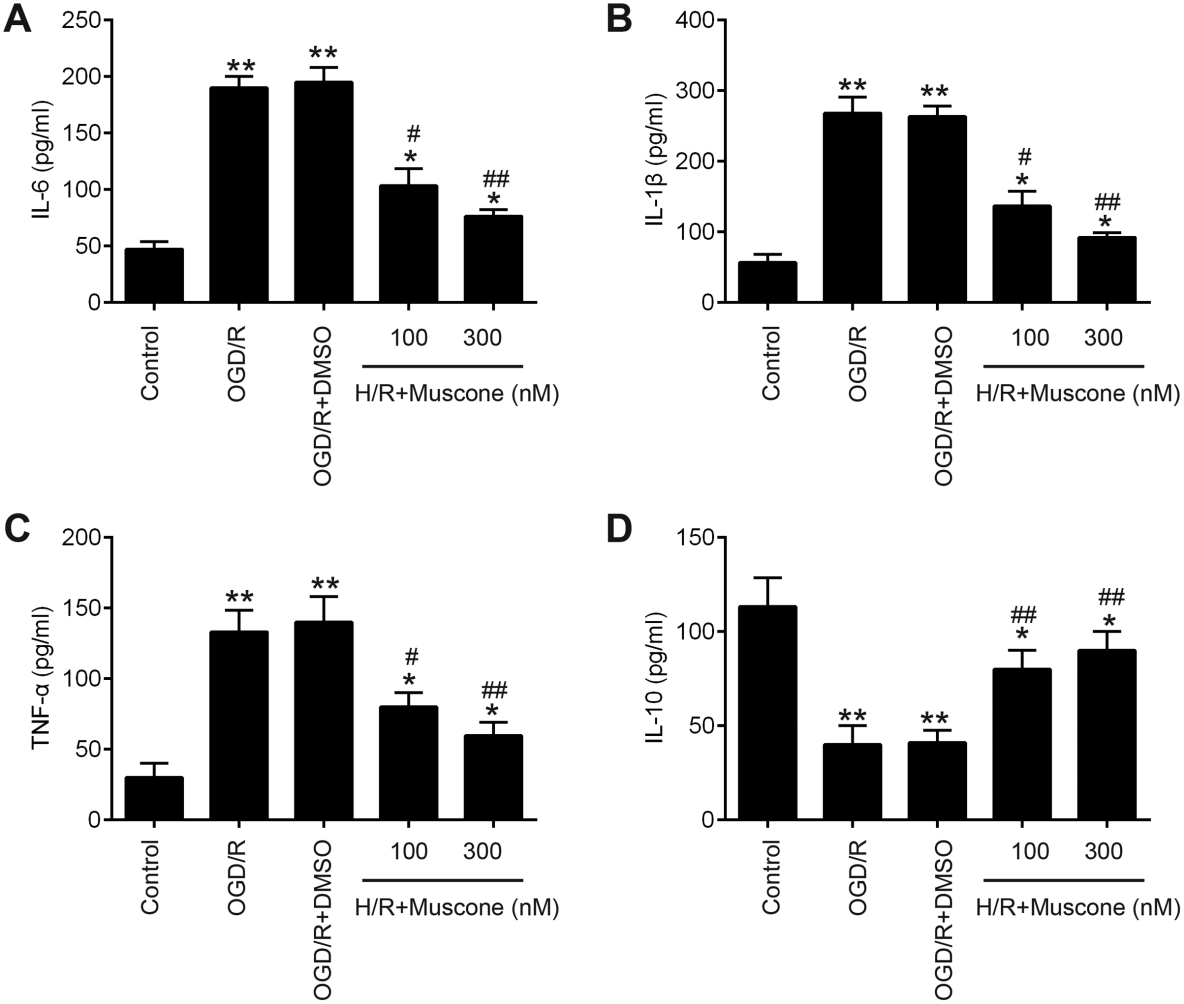
Muscone improved H/R-induced inflammatory response. HT22 cells were treated with muscone (100 nm and 300 nm) before H/R exposure. Then, cells were harvested for subsequent experiments. (A–D) The IL-6, TNF-α, IL-1β, and IL-10 concentrations were determined by ELISA kits. Data are presented as the mean ± SD of three independent experiments. *p < 0.05 and **p < 0.01 *vs*. control group; #*p* < 0.05, ##*p* < 0.01 *vs*. H/R group.

Inflammatory response is another important pathophysiologic process of cerebral hypoxia injury (Lee et al., 2018; Millar et al., 2017). Therefore, we determined whether muscone affects the inflammatory response in injured HT22 neurons. The results of ELISA assays revealed that H/R exposure resulted in a significant elevation of pro-inflammatory factors including IL-6, IL-1β and TNF-α, and a marked reduction of anti-inflammatory factor, IL-10 compared with the control group. In contrast, muscone significantly reduced the levels of these pro-inflammatory factors and enhanced the levels of anti-inflammatory factor compared with H/R group (Figs. 2A–2D; F = 115.3, df = 4, *p* < 0.01; F = 105.6, df = 4, *p* < 0.01; F = 39.72, df = 4, *p* < 0.01; F = 26.53, df = 4, *p* < 0.01). Collectively, these data indicate that muscone improve H/R-induced HT22 neurons injury by reducing inflammatory response.

### miR-142-5p was upregulated by muscone in HT22 neurons in response to H/R treatment

**Figure 3:**
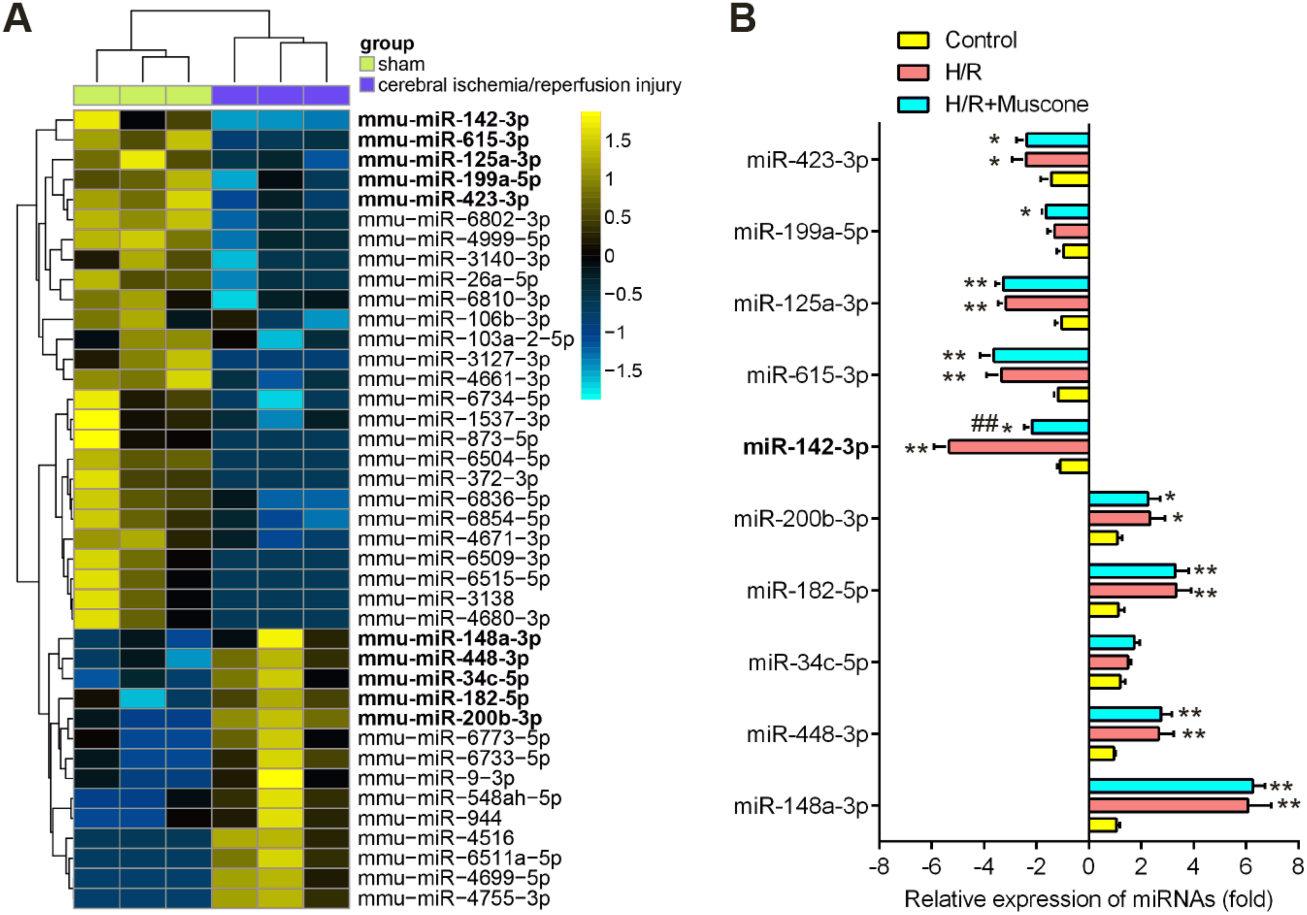
Muscone increased the expression of miR-142 in H/R-injured HT22 cells. (A) Data were retrieved from the Gene Expression Omnibus (GEO) dataset, with the accession number GSE96985 and differentially expressed miRNAs were analyzed using the “limma” package. The color code in the heat map is linear and the expression levels of miRNAs that were upregulated are shown in green to red, whereas the miRNAs that were downregulated are shown from red to green. (B) HT22 cells were treated with muscone before H/R exposure. Then cells were harvested for subsequent experiments. miR-423-3p, miR-199a-5p, miR-615-3p, miR-125a-3p, miR-142-5p, miR-200b-3p, miR-182-5p, miR-34c-5p, miR-448-3p and miR-148a-3p were further analyzed using qRT-PCR. Date are presented as the mean ± SD of three independent experiments. *p < 0.05 and **p < 0.01 *vs*. control group; ##*p* < 0.01 *vs*. H/R group.

Recently, many Chinese medicinal herbs were demonstrated to exert their potential effects through modulating miRNA expression profiles (Chu et al., 2019). To determine whether muscone has similar function, we analyzed the gene expression datasets of GSE84216 retrieved from GEO. A total of 40 miRNAs that were differentially expressed between cerebral injury and Sham group were observed (Fig. 3A). In this dataset, miR-142-5p, miR-423-3p, miR-199a-5p, miR-615-3p, and miR-125a-3p were significantly decreased, while miR-200b-3p, miR-182-5p, miR-34c-5p, miR-448-3p and miR-148a-3p were markedly increased, which are consistent with previous studies, suggesting the reliability of this dataset. Notably, RT-qPCR analysis revealed that muscone treatment significantly reversed the decreased expression of miR-142-5p caused by cerebral hypoxia injury in H/R-injured HT22 cells, while muscone exerted no impacts on the expressions of other miRNAs (Fig. 3B; F = 32.3, df = 2, *p* < 0.01). miR-142-5p was reported to have significant implications in multi-organ injuries, such as myocardial injury and hepatic injury, and its upregulation could improve these injuries (Li et al., 2020; Wang et al., 2016). Therefore, we proposed that muscone may improve H/R-induced HT22 cell injury by upregulating miR-142-5p expression.

### miR-142-5p knockdown abrogates the protective effects of muscone against H/R-induced HT22 cell injury

**Figure 4:**
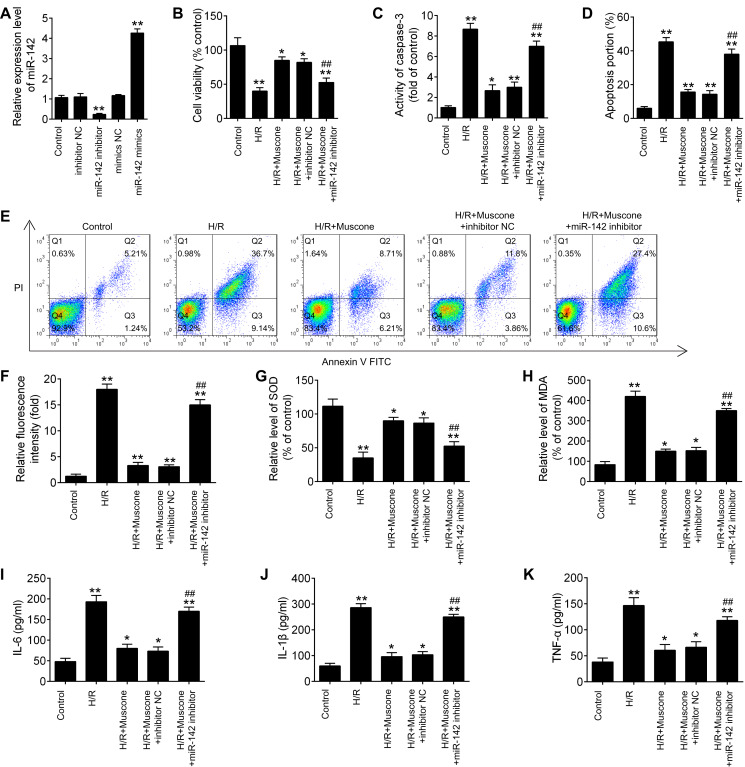
miR-142-5p knockdown abrogates the neuroprotective effects of muscone against H/R-induced HT22 cell injury. HT22 cells were pre‑transfected with miR-142-5p inhibitor or inhibitor NC for 30 min and then treated with muscone before H/R exposure. Then cells were harvested for subsequent experiments. (A) The expression levels of miR-142-5p was measured by qRT-PCR after miR-142-5p inhibitor alone transfection. (B) The cell viability of each group was determined by CCK‑8 assay. (C) The activity of caspase was measured by Caspase 3 Activity kit. (D) ROS production was detected by DCFH-DA assay. (E, F) The levels of SOD and MDA were assessed using commercial kits. (G–I) The IL-6, IL-1β and TNF-α concentrations were determined by ELISA kits. Data are presented as the mean ± SD of three independent experiments. *p < 0.05 and **p < 0.01 *vs*. control group; ##*p* < 0.01 *vs*. H/R + mu scone + inhibitor NC group.

To further explore the role of miR-142-5p in muscone-induced neuroprotection, miR-142-5p expression was knocked down in the HT22 cells by miR-142-5p inhibitor transfection. The results of RT-qPCR analysis revealed that miR-142-5p expression was notably reduced after miR-142-5p inhibitor transfection (Fig. 4A; F = 89.6, df = 4, *p* < 0.01). First, the effects of muscone on control cells were assessed and the results showed that muscone (100 nM and 300 nM) had no influence on the cell viability, the activity of caspase 3, ROS production and inflammation in HT22 cells (Fig. S1). Moreover, we found that transfection of miR-142-5p inhibitor alone led to cell damage similarly to the H/R-induced result (Fig. S1), suggesting miR-142-5p may play an important role in H/R induced HT22 cell damage. Meanwhile, we also explore the functional effects of miR-142-5p knockdown on H/R-induced HT22 cell injury. It was shown that the H/R + miR142 inhibitor group exhibited much lower cell viability, higher activity of caspase 3 and ROS production, lower levels of SOD, higher levels of MDA, as well as higher IL-6, TNF-α, IL-1β and lower IL-10 concentrations in comparison with the H/R + inhibitor group, suggesting that miR-142-5p knockdown could aggravate H/R-induced HT22 cell injury (Fig. S2). Subsequently, we found that miR-142-5p knockdown reversed the muscone-induced upregulation of cell viability in the H/R-injured HT22 cells (Fig. 4B; F = 42.46, df = 4, *p* < 0.01). In addition, the effects of miR-142-5p on the activity of caspase 3 in the presence of muscone under H/R conditions were also investigated. As shown in Fig. 4C, miR-142-5p knockdown attenuated the muscone-induced inhibition on the activity of caspase 3 under H/R conditions (F = 29.9, df = 4, *p* < 0.01). Meanwhile, we also found that transfection of miR-142-5p significantly weakened the inhibitory effect of muscone on cell apoptosis (Figs. 4D and 4E; F = 185.5, df = 4, *p* < 0.01). Next, the roles of miR-142-5 in the effects of muscone on oxidative stress were further examined in H/R-injured HT22 cells. It was shown that H/R-induced increase in ROS levels was attenuated by muscone treatment; however, the inhibitory effects of muscone was abolished by miR-142-5 knockdown (Fig. 4F; F = 43.3, df = 4, *p* < 0.01). Meanwhile, the H/R-induced decrease in SOD levels were reversed by muscone treatment, and the increased MDA levels were attenuated by muscone treatment; however, these effects of muscone were eliminated by miR-142-5 knockdown (Figs. 4G and 4H; F = 46.32, df = 4, *p* < 0.01; F = 31.3, df = 4, *p* < 0.01). We also examined the effects of miR-142-5p knockdown on the inflammatory response in the presence of muscone under H/R conditions. As expected, the increased expression levels of IL-6, IL-1β and TNF-α caused by H/R were attenuated by muscone; however, these inhibitory effects of muscone were also reversed by miR-142-5p knockdown (Figs. 4I–4K; F = 63.4, df = 4, *p* < 0.01; F = 88.0, df = 4, *p* < 0.01; F = 52.27, df = 4, *p* < 0.01). Collective, these data suggest that miR-142-5p mediated the neuroprotective effects of muscone against H/R-induced HT22 cell injury.

### HMGB1 is a direct target of miR-142-5p

**Figure 5:**
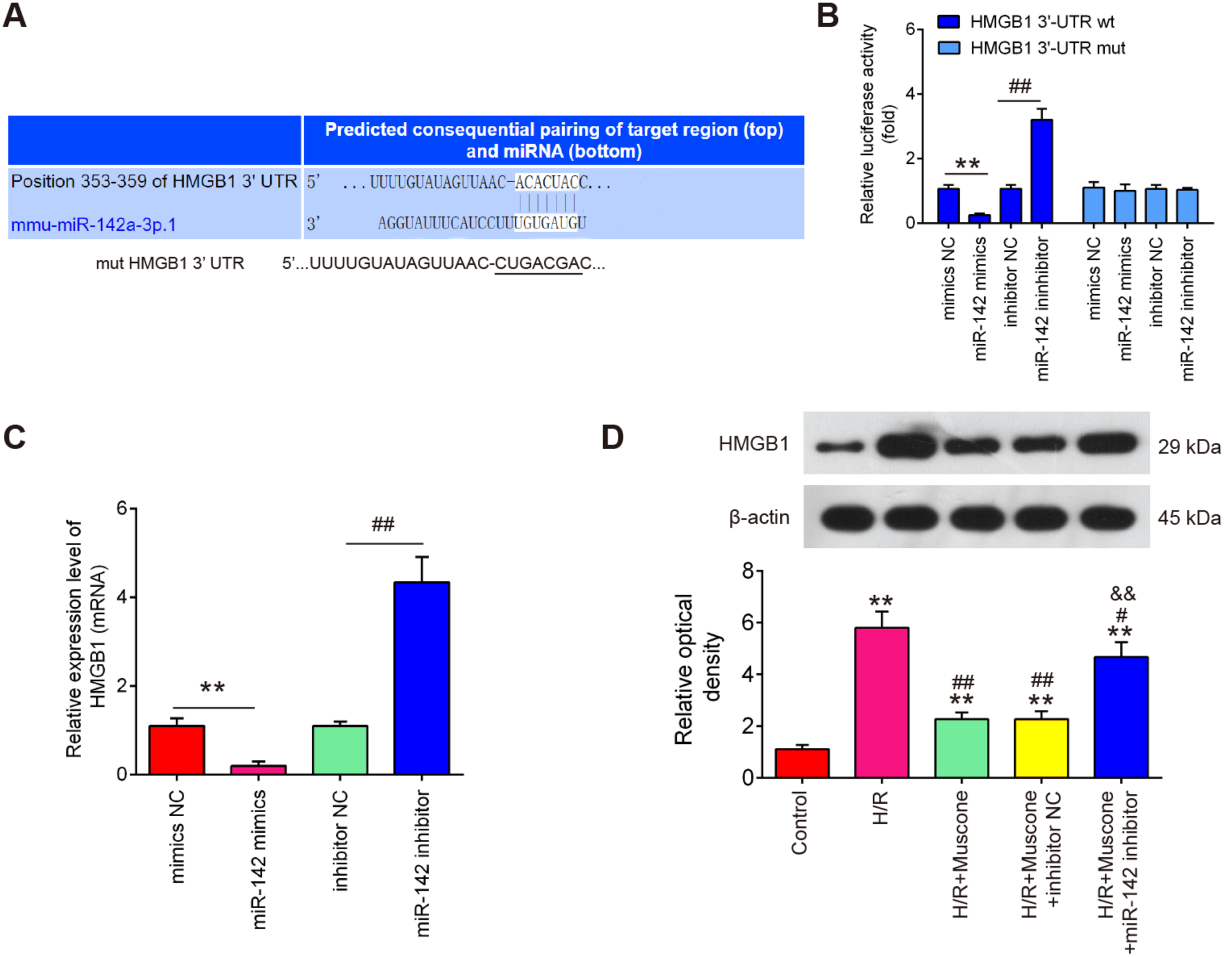
HMGB1 is a direct target of miR-142-5p. (A) The predicted complementary sequences for miR-142-5p in the 3′-UTR of HMGB1 and the mutations are shown in the seed region of miR-142-5p. (B) The HT22 cells were co-transfected with either pmirGLO-HMGB1-3′-UTR or pmirGLO-HMGB1-mut-3′-UTR, and miR-142-5p mimics/inhibitor or corresponding NC and the relative luciferase activity were measured. **p < 0.01 *vs*. mimic NC. ##*p* < 0.01 *vs*. inhibitor NC. (C) The HT22 cells were transfected with miR-142-5p mimics/inhibitor or corresponding NC, and the HMGB1 protein level was measured using Western blot analysis. β-actin was used as an internal control. (D) HT22 cells were pre‑transfected with miR-142-5p inhibitor or inhibitor NC for 30 min and then treated with muscone before H/R exposure. Then cells were harvested for subsequent experiments. HMGB1 protein level was measured using Western blot analysis. Data are presented as the mean ± SD of three independent experiments. **p < 0.01 *vs*. control group; #*p* < 0.05, ##*p* < 0.01 *vs*. H/R; &&*p* < 0.01 *vs*. H/R + muscone group.

To further evaluate the mechanisms by which miR-142-5p mediates the neuroprotective effects of muscone under H/R conditions, miRNA targets were investigated using two bioinformatic tools (Targetscan and miRBase). As shown in Fig. 5A, miR-142-5p was predicted as a putative miRNA targeting HMGB1, a well-known pro-inflammatory mediator. To examine if miR-142-5p directly target HMGB1, a luciferase reporter assay was performed. It was shown that overexpression of miR-142-5p significantly reduced, whereas miR-142 inhibition increased the relative luciferase activity of HMGB1 3′-UTR wt. However, no obvious changes in the luciferase activity were found when HT22 cells were co-transfected with HGMB1-3′-UTR mut reporter and miR-142-5p mimics/miR-142 inhibitor (Fig. 5B). RT-qPCR analysis showed that HGMB1 mRNA expression was significantly down-regulated after miR-142-5p mimics transfection and increased by miR-142 inhibitor in HT22 cells (Fig. 5C). We also found that H/R exposure resulted in a significant increase in the expression of HGMB1 protein, compared with control group, while the increased expression of HGMB1 protein was attenuated by muscone treatment. Moreover, the inhibitory effect of muscone was reversed by miR-142-5p knockdown (Fig. 5D; F = 62.07, df = 4, *p* < 0.01). These data suggest that HMGB1 is a direct target of miR-142-5p in H/R-induced HT22 cells.

### Muscone inactivates the TLR4/NF-κB signaling pathway in H/R-injured HT22 cells

**Figure 6:**
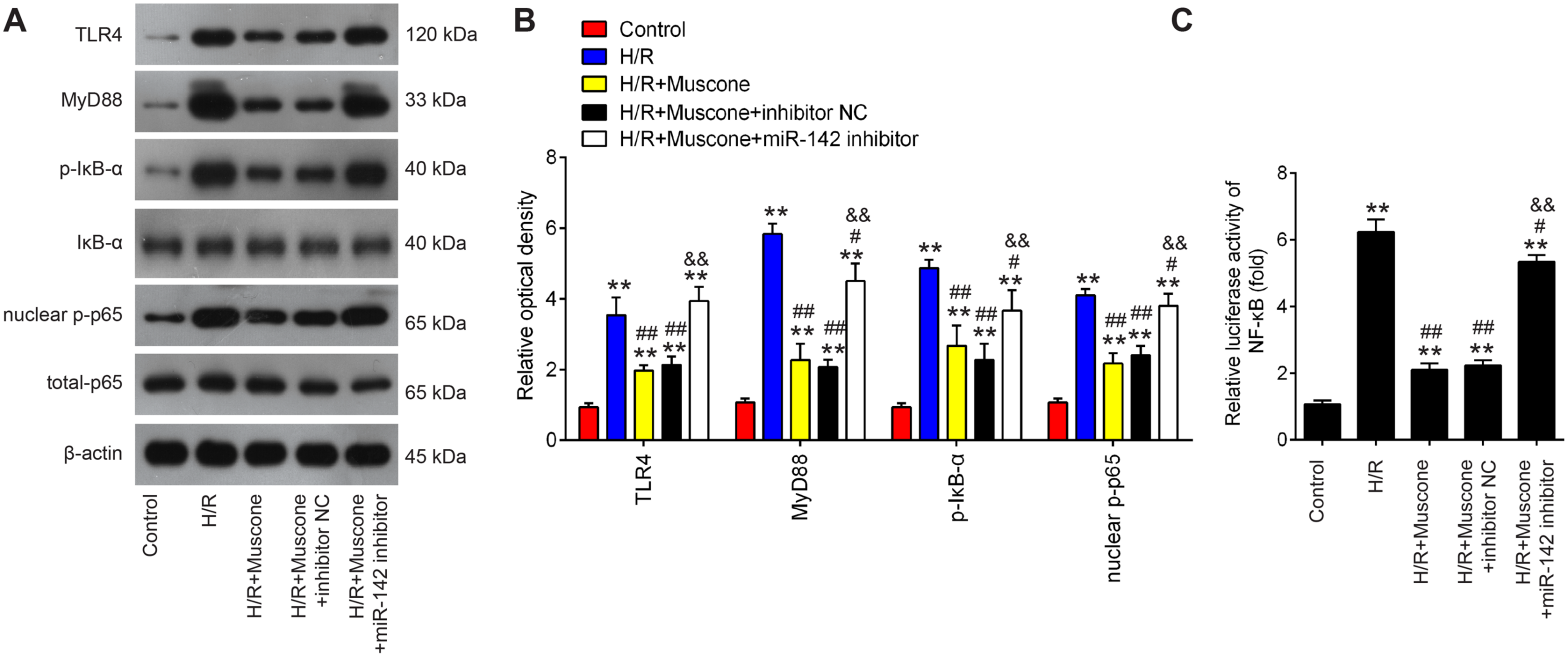
Muscone inactivates the TLR4/NF-κB signaling pathway in H/R-injured HT22 cells. HT22 cells were pre-transfected with miR-142-5p inhibitor or inhibitor NC for 30 min and then treated with muscone before H/R exposure. Then cells were harvested for subsequent experiments. (A) Protein levels of TLR4, MyD88, p-IκBα and nuclear p-p65 were detected by western blot analysis. (B) The bands were semi-quantitatively analyzed by using ImageJ software. (C) NF-κB activity was assessed using the NF-κB activity assay. Data are presented as the mean ± SD of three independent experiments. **p < 0.01 *vs*. control group; #*p* < 0.05 and ##*p* < 0.01 *vs*. H/R; &&*p* < 0.01 *vs*. H/R + muscone group.

The TLR4/NF-κB pathway is known to be involved in neuronal inflammatory response and oxidative stress associated with cerebral injury (Chen et al., 2020; Zhang et al., 2019). Since HMGB1 is an important mediator for efficient induction of TLR4/NF-κB pathway, it was proposed that this pathway maybe involved in the neuroprotective effects of muscone under H/R conditions. It was shown that TLR4, MyD88, p-IκBα and nuclear p-p65 were significantly increased in HT22 cells exposed to H/R, indicating that H/R activated TLR4/NF-κB signaling pathway. However, these effects were reversed by muscone treatment (Figs. 6A and 6B). It was also observed that the inhibitory effects of muscone on these protein expressions were attenuated by miR-142-5p knockdown in HT22 cells under H/R conditions (Figs. 6A and 6B). To further confirm the association between miR-142-5p and TLR4/NF-kB signaling pathway, the NF-κB activity assay was performed. As shown in Fig. 6C, the NF-κB activity was significantly increased under H/R stimulation, whereas it was attenuated after muscone treatment. As expected, the inhibitory effects of muscone on the NF-κB activity was reversed by miR-142-5p knockdown (F = 88.5, df = 4, *p* < 0.01). All these data suggest that Muscone inactivates the TLR4/NF-κB signaling pathway through regulating miR-142-5p expression in H/R-injured HT22 cells.

## Discussion

In the present study, muscone protected HT22 cells against H/R injury by suppressing apoptosis, oxidative stress and inflammatory response. Mechanistically, our findings showed that muscone upregulated the expression levels of miR-142-5p, subsequently decreased expression of HGMB1 by binding to 3′-UTR region of HGMB1 mRNA, leading to the inactivation of TLR4/NF-κB pathway, thereby exerting protective effects against H/R-induced HT22 cell injury. These findings suggest that muscone may be used as a candidate agent to treat cerebral hypoxia injury.

In recent years, the progress in research on neuroprotective effects of traditional Chinese medicine has been increasingly investigated in brain injury (Cheng & Lee, 2016; Feigin, 2007; Ip et al., 2016). Muscone, a ventral glandular secretion of the male musk deer obtained from musk, has been extensively studies in animal models of cardiovascular and cerebrovascular disorders due to its anti-oxidant, anti-inflammatory and anti-apoptotic properties (Liang et al., 2010; Wu et al., 2011; Zhang et al., 2007). Notably, muscone exerted neuroprotection in middle cerebral artery occluded (MCAO) rats, as reflected by the reduction of cerebral infarct volume, neurological dysfunction and inhibition of cortical neuron apoptosis (Wei et al., 2012). The results in this study revealed that muscone markedly reduced cell apoptosis, oxidative stress and inflammatory response in H/R-injured HT22 cells. These results suggested that muscone may improve H/R-induced injury in HT22 cells. However, the underlying mechanisms of the neuroprotective effects of muscone have not yet been elucidated.

Numerous studies have shown that miRNAs are involved in the modulation function of traditional Chinese medicine (TCM) in brain injury (Bian, Shan & Chen, 2017; Ma et al., 2020). For example, *Rhodiola crenulata* attenuated hypobaric hypoxia (HH)-induced brain injury by regulating apoptosis and mitochondrial energy metabolism *via* the miR-210/ISCU1/2 (COX10) signaling pathway (Wang et al., 2019b). Chu et al. (2019) showed that Ginsenoside Rg1 (Rg1) protected PC12 cells against neuronal injury by alleviating oxidative stress through inhibiting miR-144 activity. Yang et al. (2021) found that Gualou Guizhi Decoction (GLGZD) down-regulated miR-155, mediating subsequent neuroinflammation and resulting in neuroprotection in MCAO rats. Wang et al. (2019a) reported that Trametenolic acid B (TAB) could efficiently improve learning and memory ability of injured rats and suppress mitochondrial-mediated neuron apoptosis through modulation of miR-10a. Nevertheless, whether miRNAs are involved the improvement of muscone in H/R-injured HT22 cells remains unclear. In this study, 40 differentially expressed-miRNAs were observed between cerebral injury and Sham group through retrieving the gene expression datasets GSE84216. Notably, treatment with muscone was identified to cause miR-142-5p upregulation in injured HT22 cells. A recent study showed that miR-142-3p were identified as markers after traumatic brain injury that could be useful for distinguishing severity and improvement over time (Mitra et al., 2017). Another study has shown that down-regulation of miR-142-5p could attenuate oxygen-glucose deprivation and reoxygenation (OGD/R)-induced neuron injury through promoting Nrf2 expression (Wang et al., 2017). However, whether miR-142-5p contributed to the protective effects of muscone on hypoxia injury remain unknown. In the present study, our results revealed that miR-142 inhibition abolished the neuroprotective effects of muscone *in vitro*, suggesting that miR-142 upregulation contributes to the neuroprotective effects of muscone in H/R induced cell injury.

High mobility group protein B1 (HMGB1) is a highly conserved DNA-binding protein that is locate in the nucleus of mammalian cells. During hypoxia injury, HMGB1, as an endogenous ligand of Toll-like receptors 4 (TLR4), is secreted into the cytoplasm (Liu et al., 2016), and the extracellular HMGB1 can stimulate TLR4/NF-κB pathway, thereby triggers the release of ROS and pro-inflammatory cytokines including TNF-α, IL-6 and IL-1β, which further enhances inflammatory response and causes tissue damage (van Beijnum, Buurman & Griffioen, 2008; Zhang et al., 2017). Previous studies have demonstrated that suppression of HMGB1/TLR4/NF-κB pathway could improve cerebral injury in mice (Cheng et al., 2017; Tang et al., 2007). For example, Zhai et al. (2020) found that dexmedetomidine (Dex) treatment can alleviate cerebral injury in rats by inhibiting the HMGB1/TLR4/NF-κB signaling pathway. Xie et al. (2019) showed that inhibiting HMGB1/TLR4/NF-κB pathway triggered inflammation was associated with neuroprotective effects of Notoginseng leaf triterpenes (PNGL) against cerebral injury. Therefore, whether the protective role of muscone against H/R injury was associated with HMGB1/TLR4/NF-κB pathway attracted our attention. In our study, HMGB1 was confirmed as a direct target of miR-142-5p, which is consistent with a previous study (Jiang et al., 2017). Therefore, we hypothesized that muscone may exhibit its neuroprotective effect against cerebral hypoxia injury through inhibiting HMGB1-mediated TLR4/NF-κB pathway. Based on western blot, we found that H/R stimulation led to increased expression levels of TLR4, MyD88, p-IκBα and nuclear p-p65; however, these effects can be attenuated by muscone, which was antagonized by miR-142-5p knockdown, suggesting that muscone effectively inhibited activation of HMGB1 mediated TLR-4/NF-κB signaling pathway by upregulating miR-142-5p expression.

However, there are some limitations of our study. First, we use the HT-22 cells which possess glycolytic phenotype as a neuronal model, therefore, we will explore the effects of muscone on the level of cell viability, ROS and apoptotic markers in primary neuronal cell cultures. Second, we used oxygen deprivation (95% N_2_ and 5% CO_2_) condition in the beginning, but there was more shedding of cells. Thus, we finally changed the cell culture condition to hypoxia/reoxygenation (92% N_2_, 3% O_2_ and 5% CO_2_). Although this approach has also been reported in the literatures, we will further using oxygen deprivation/reoxygenation model in future. Third, cell death process is a dynamic event with subsequent activation of particular processes and neuro-inflammatory response is usually a consequence of primary injury. However, only one time point was used in our study. In future, we will detect cell activity, apoptosis, inflammatory response at different time points after reoxygenation to assess the dynamic processes regulated by drugs on measured parameters. Finally, our study the differences between two experimental groups (H/R + inhibitor NC and H/R + miR142 inhibitor) in the TLR4/NF-κB signaling pathway detection were not evaluated; thus, data interpretation could be somehow affected, and further studies are warranted to confirm these results.

## Conclusions

In summary, we for the first time demonstrate that muscone treatment can protect neurons against H/R-induced injury by suppressing inflammatory response, oxidative stress and apoptosis through the miR-142-5p/HMGB1/TLR-4/NF-κB pathway. All these findings suggest that muscone may be a promising neuroprotective agent of ischemic stroke damage, which warrants further research in the application of muscone in the treatment of ischemic stroke.

## Supplemental Information

10.7717/peerj.13523/supp-1 Supplemental Information 1 miR-142-5p knockdown suppressed the cell viability, promoted the inflammation, oxidative stress and the apoptosis in HT22 cells. miR-142 inhibitor and inhibitor-NC were transfected into HT22 cells, or muscone (100 and 300 nM) was added to HT22 cells. Then, cells were harvested for subsequent experiments. (A) The cell viability of each group was determined by CCK‑8 assay. (B) The activity of caspase was measured by Caspase 3 Activity kit. (C) ROS production was detected by DCFH-DA assay. (D, E) The levels of SOD and MDA were assessed using commercial kits. (F-I) The IL-6, TNF-α, IL-1β, and IL-10 concentrations were determined by ELISA kits. Data are presented as the mean ± SD of three independent experiments. *p < 0.05 and **p < 0.01 *vs*. inhibitor NC group.

10.7717/peerj.13523/supp-2 Supplemental Information 2 miR-142-5p knockdown aggravated H/R-induced HT22 cell injury. miR-142-5p inhibitor and inhibitor NC were transfected into HT22 cells, followed by H/R stimulation. Then, cells were harvested for subsequent experiments. (A) The cell viability of each group was determined by CCK‑8 assay. (B) The activity of caspase was measured by Caspase 3 Activity kit. (C) ROS production was detected by DCFH-DA assay. (D, E) The levels of SOD and MDA were assessed using commercial kits. (F-I) The IL-6, TNF-α, IL-1β, and IL-10 concentrations were determined by ELISA kits. Data are presented as the mean ± SD of three independent experiments. *p < 0.05 and **p < 0.01 *vs*. H/R + inhibitor NC group.

10.7717/peerj.13523/supp-3 Supplemental Information 3 The original WB image of Figure 5.

10.7717/peerj.13523/supp-4 Supplemental Information 4 The original WB image of Figure 6.

10.7717/peerj.13523/supp-5 Supplemental Information 5 The original microscopy or flow cytometry data.

10.7717/peerj.13523/supp-6 Supplemental Information 6 The original source data used in tables and figures.

## Additional Information and Declarations

### Data Availability

The following information was supplied regarding data availability: The raw measurements are available in the Supplemental Files.
