# Supplementary figures and images for: Muscone improves hypoxia/reoxygenation (H/R)-induced neuronal injury by blocking HMGB1/TLR4/NF-κB pathway via modulating microRNA-142
Source: PeerJ. 2022 Jul 15;10:e13523. doi: 10.7717/peerj.13523 (PMC9290999; doi:10.7717/peerj.13523)

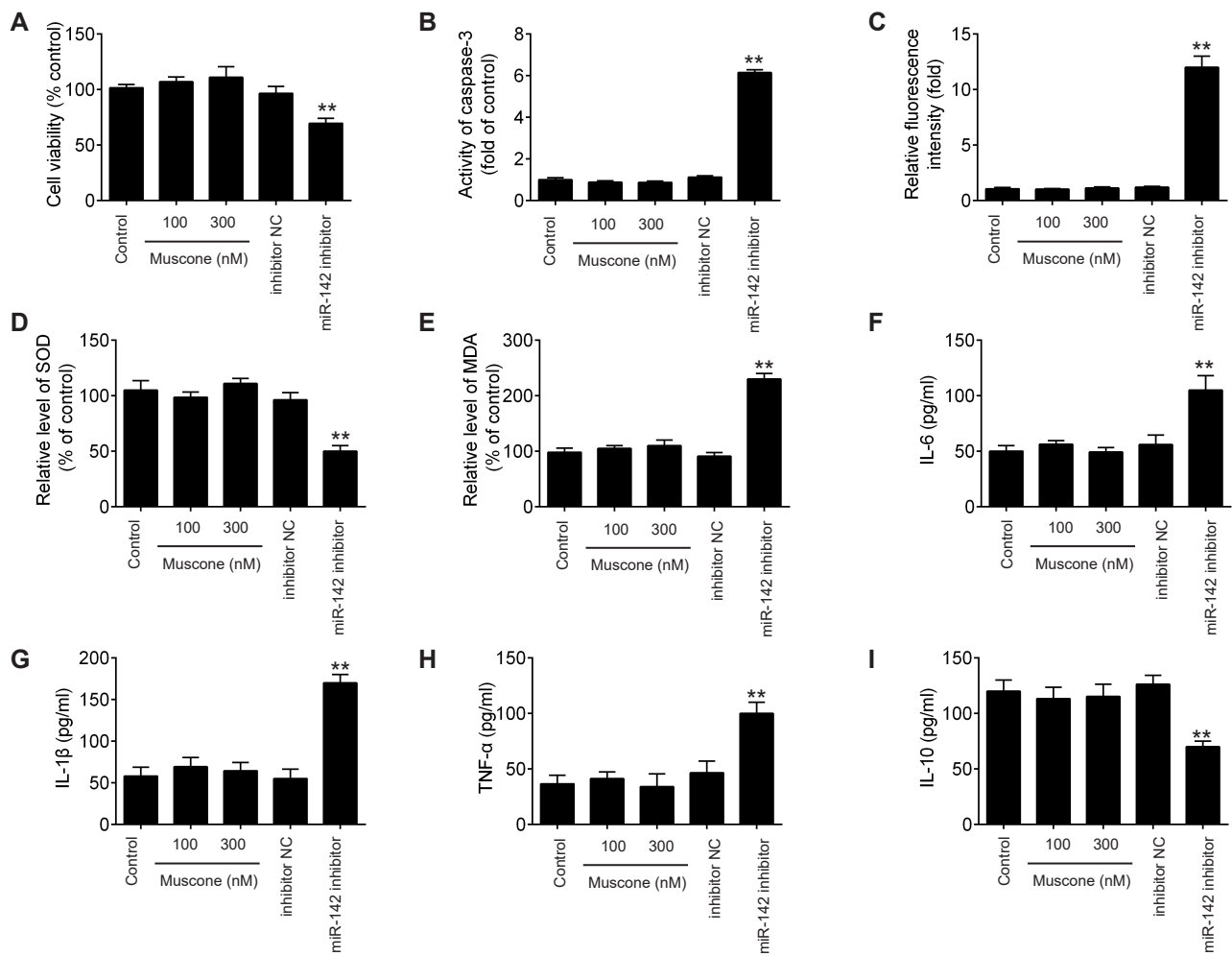

Supplement: Supplemental Information 1 — miR-142 inhibitor and inhibitor-NC were transfected into HT22 cells, or muscone (100 and 300 nM) was added to HT22 cells. Then, cells were harvested for subsequent experiments. (A) The cell viability of each group was determined by CCK‑8 assay. (B) The activity of caspase was measured by Caspase 3 Activity kit. (C) ROS production was detected by DCFH-DA assay. (D, E) The levels of SOD and MDA were assessed using commercial kits. (F-I) The IL-6, TNF-α, IL-1β, and IL-10 concentrations were determined by ELISA kits. Data are presented as the mean ± SD of three independent experiments. *p < 0.05 and **p < 0.01 vs. inhibitor NC group. [file peerj-10-13523-s001.pdf]

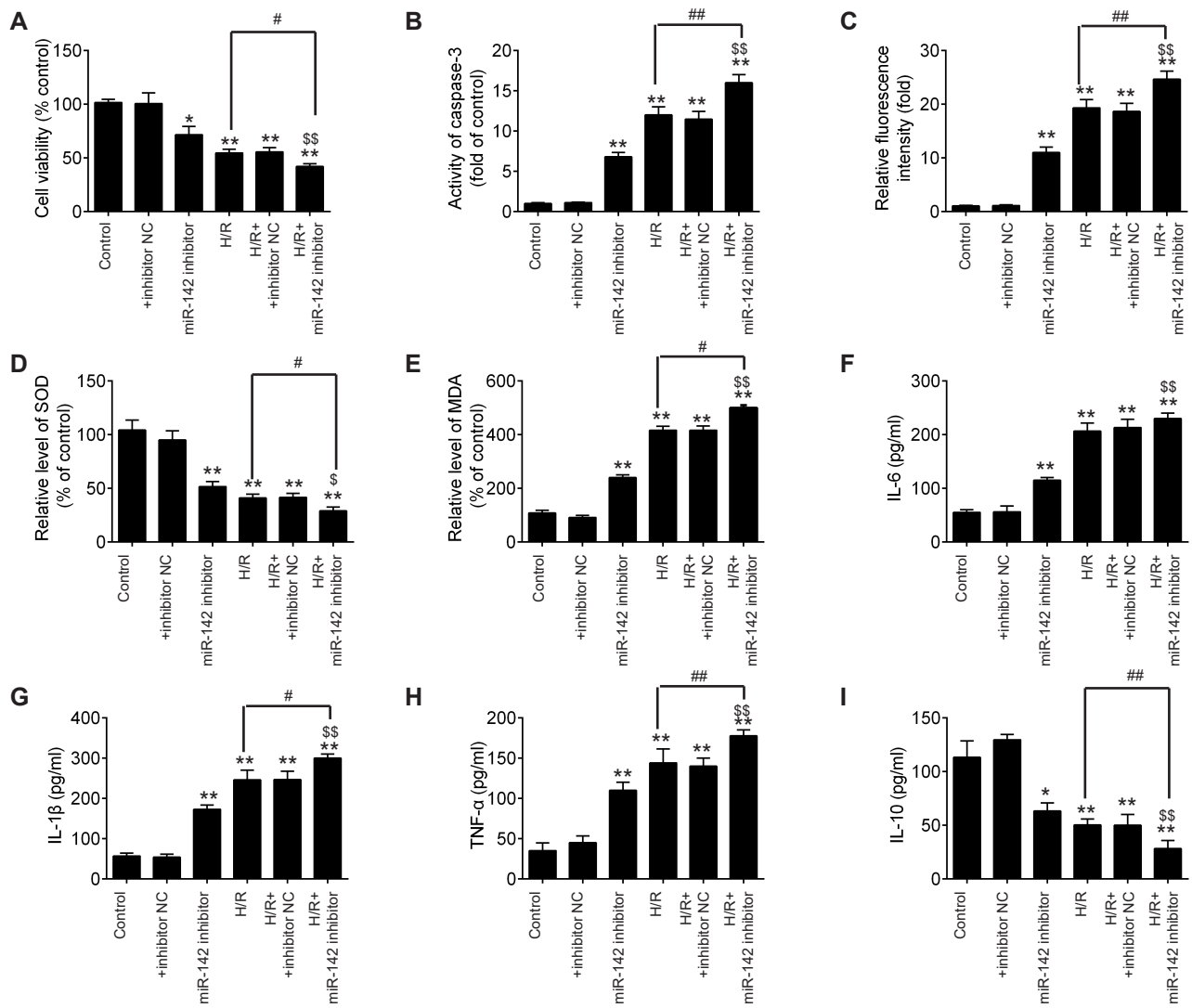

Supplement: Supplemental Information 2 — miR-142-5p inhibitor and inhibitor NC were transfected into HT22 cells, followed by H/R stimulation. Then, cells were harvested for subsequent experiments. (A) The cell viability of each group was determined by CCK‑8 assay. (B) The activity of caspase was measured by Caspase 3 Activity kit. (C) ROS production was detected by DCFH-DA assay. (D, E) The levels of SOD and MDA were assessed using commercial kits. (F-I) The IL-6, TNF-α, IL-1β, and IL-10 concentrations were determined by ELISA kits. Data are presented as the mean ± SD of three independent experiments. *p < 0.05 and **p < 0.01 vs. H/R + inhibitor NC group. [file peerj-10-13523-s002.pdf]

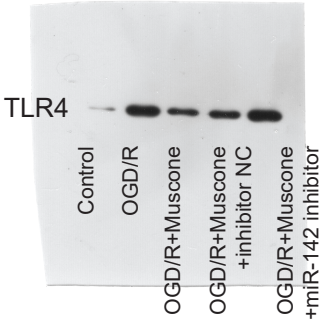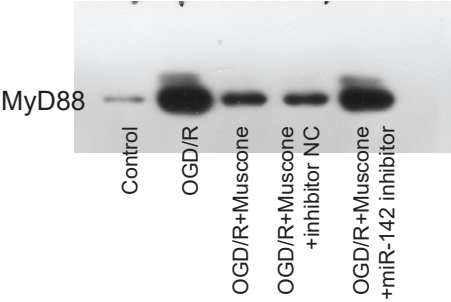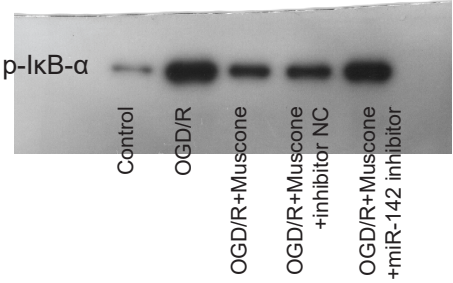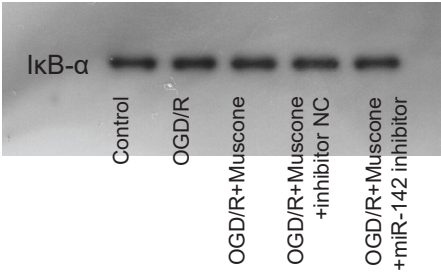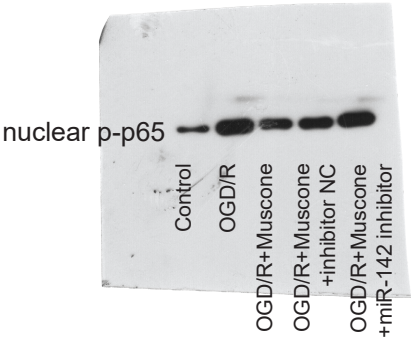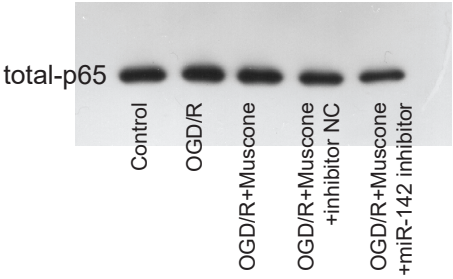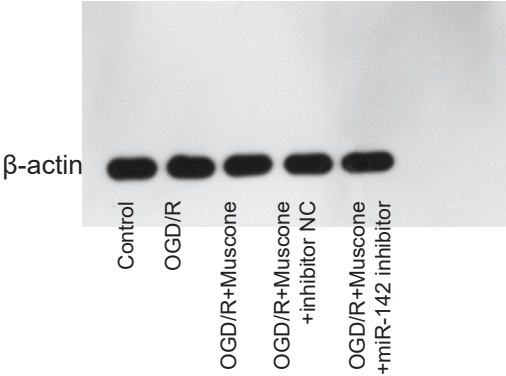

Supplement: Supplemental Information 4 [file peerj-10-13523-s004.pdf]

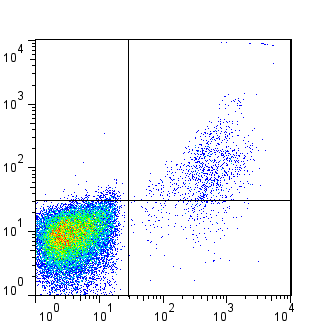

Supplement: Supplemental Information 5 [file peerj-10-13523-s005.zip › 2 the original files for any microscopy or flow cytometry data/flow cytometry data/Figure 1D/Apoptosis-1.tif]

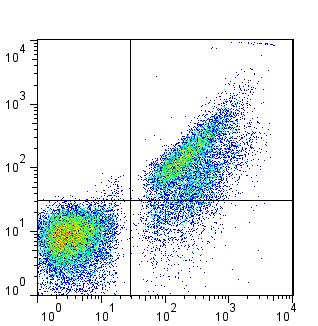

Supplement: Supplemental Information 5 [file peerj-10-13523-s005.zip › 2 the original files for any microscopy or flow cytometry data/flow cytometry data/Figure 1D/Apoptosis-2.tif]

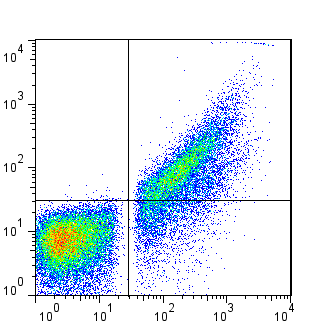

Supplement: Supplemental Information 5 [file peerj-10-13523-s005.zip › 2 the original files for any microscopy or flow cytometry data/flow cytometry data/Figure 1D/Apoptosis-3.tif]

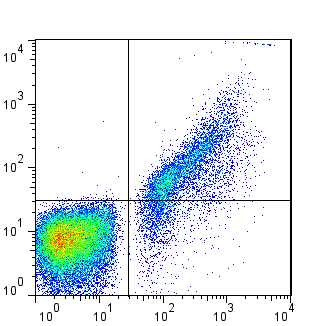

Supplement: Supplemental Information 5 [file peerj-10-13523-s005.zip › 2 the original files for any microscopy or flow cytometry data/flow cytometry data/Figure 1D/Apoptosis-4.tif]

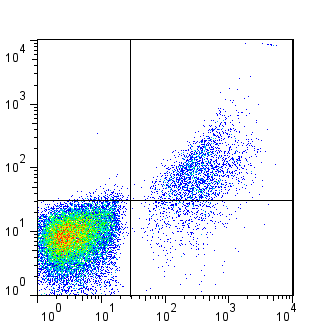

Supplement: Supplemental Information 5 [file peerj-10-13523-s005.zip › 2 the original files for any microscopy or flow cytometry data/flow cytometry data/Figure 1D/Apoptosis-5.tif]

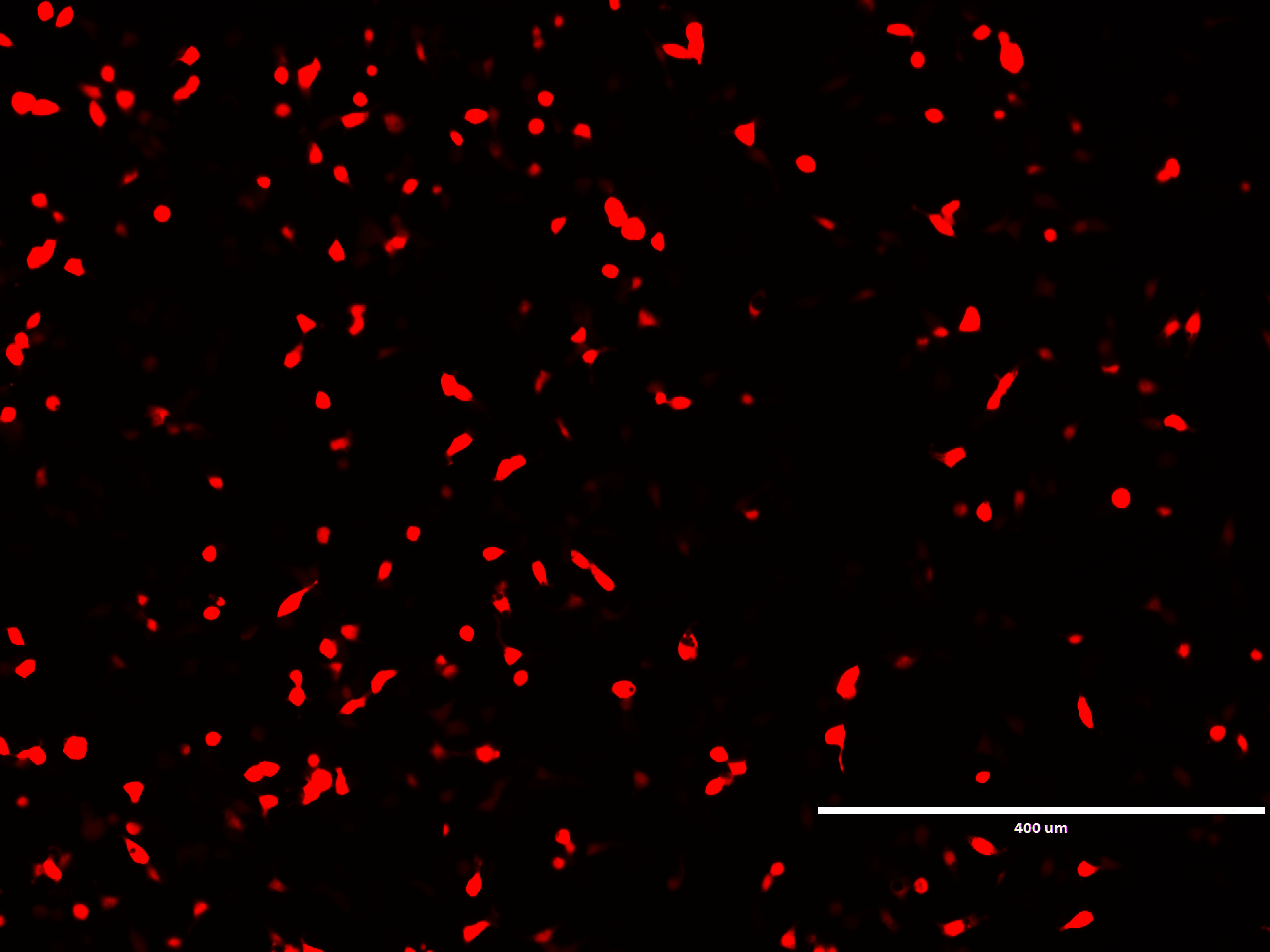

Supplement: Supplemental Information 5 [file peerj-10-13523-s005.zip › 2 the original files for any microscopy or flow cytometry data/microscopy data/Figure 1E/OGDR+DMSO.tif]

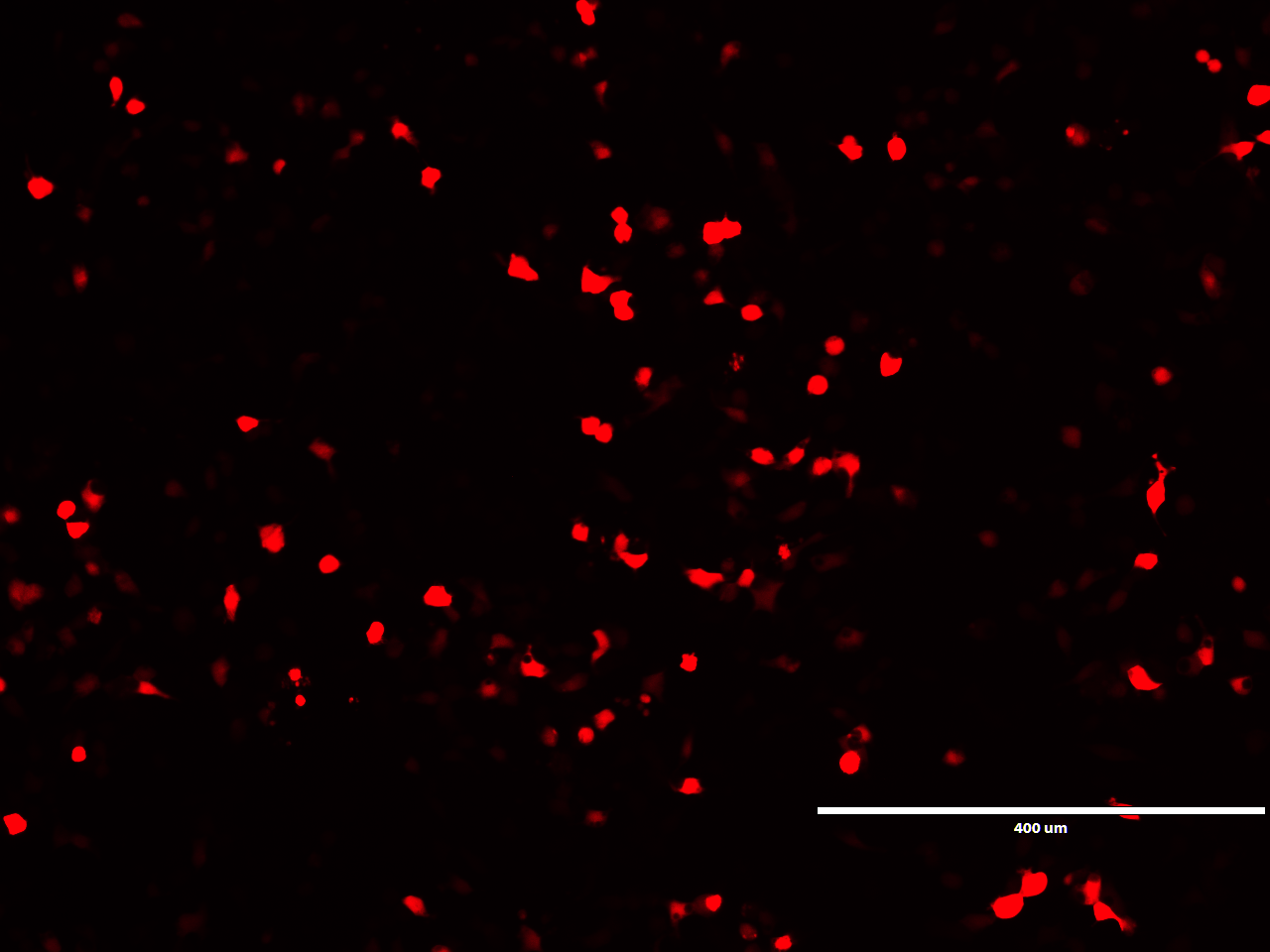

Supplement: Supplemental Information 5 [file peerj-10-13523-s005.zip › 2 the original files for any microscopy or flow cytometry data/microscopy data/Figure 1E/OGDR+Muscone100ng.tif]

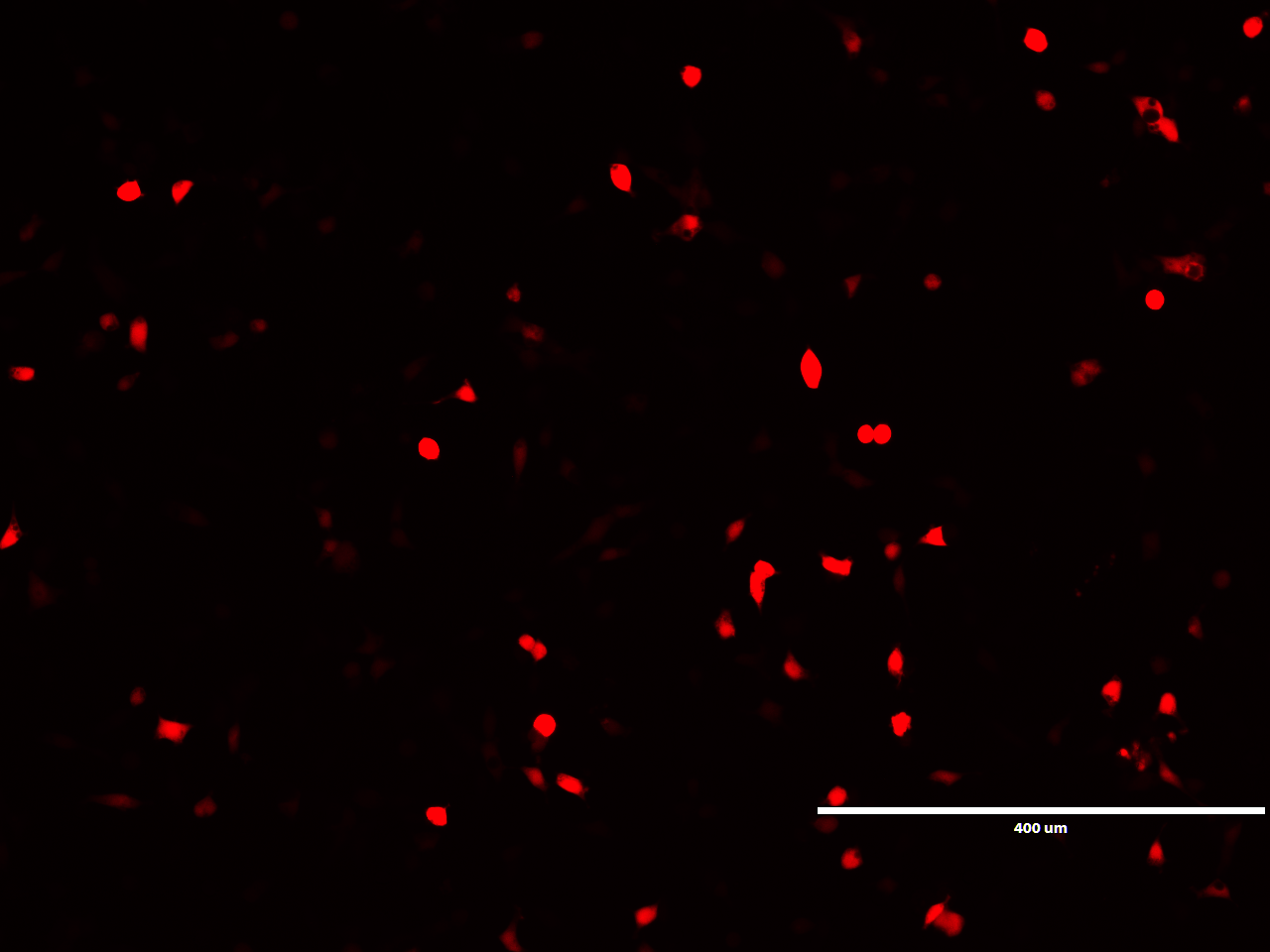

Supplement: Supplemental Information 5 [file peerj-10-13523-s005.zip › 2 the original files for any microscopy or flow cytometry data/microscopy data/Figure 1E/OGDR+Muscone300ng.tif]

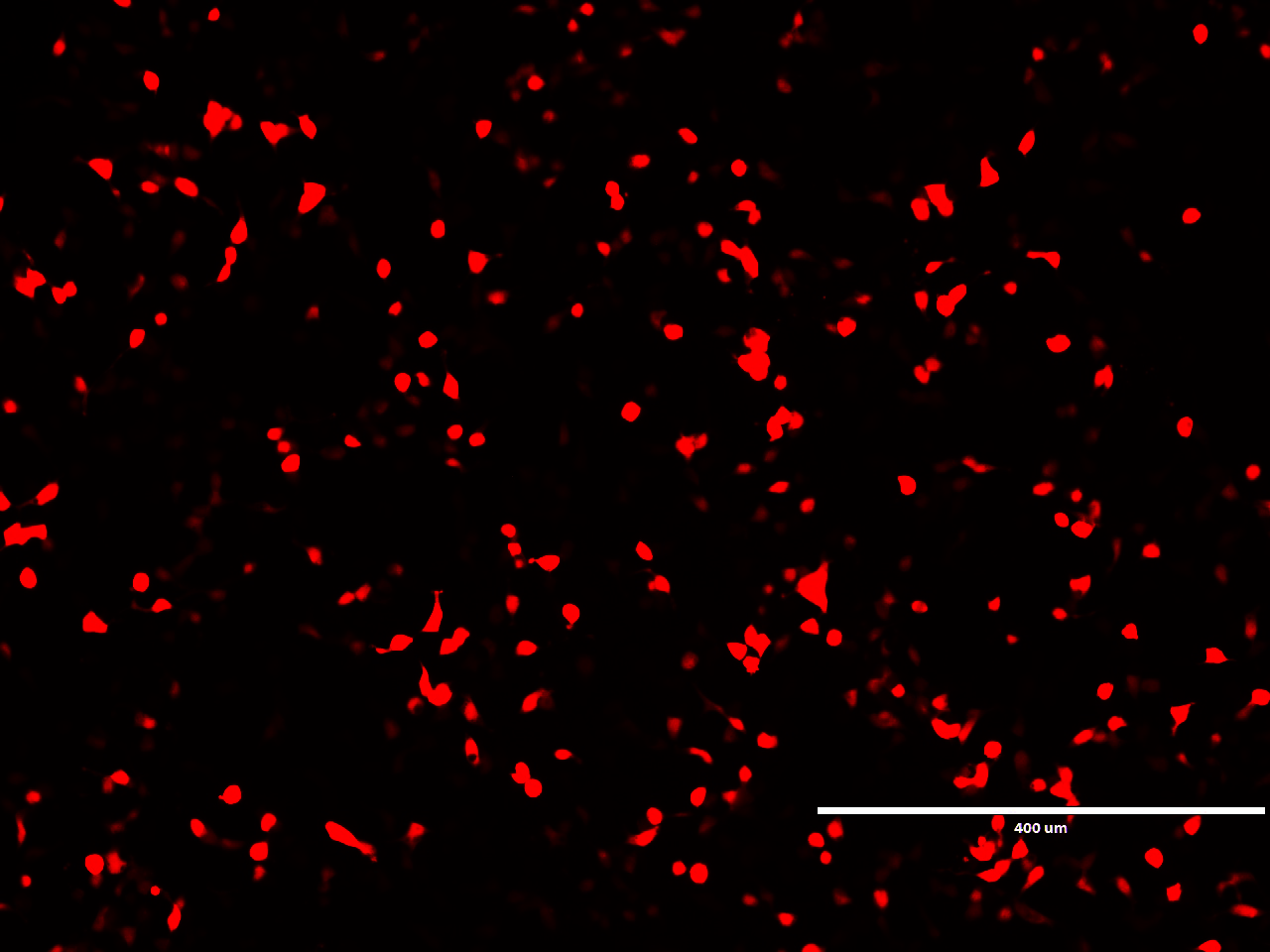

Supplement: Supplemental Information 5 [file peerj-10-13523-s005.zip › 2 the original files for any microscopy or flow cytometry data/microscopy data/Figure 1E/OGDR.tif]

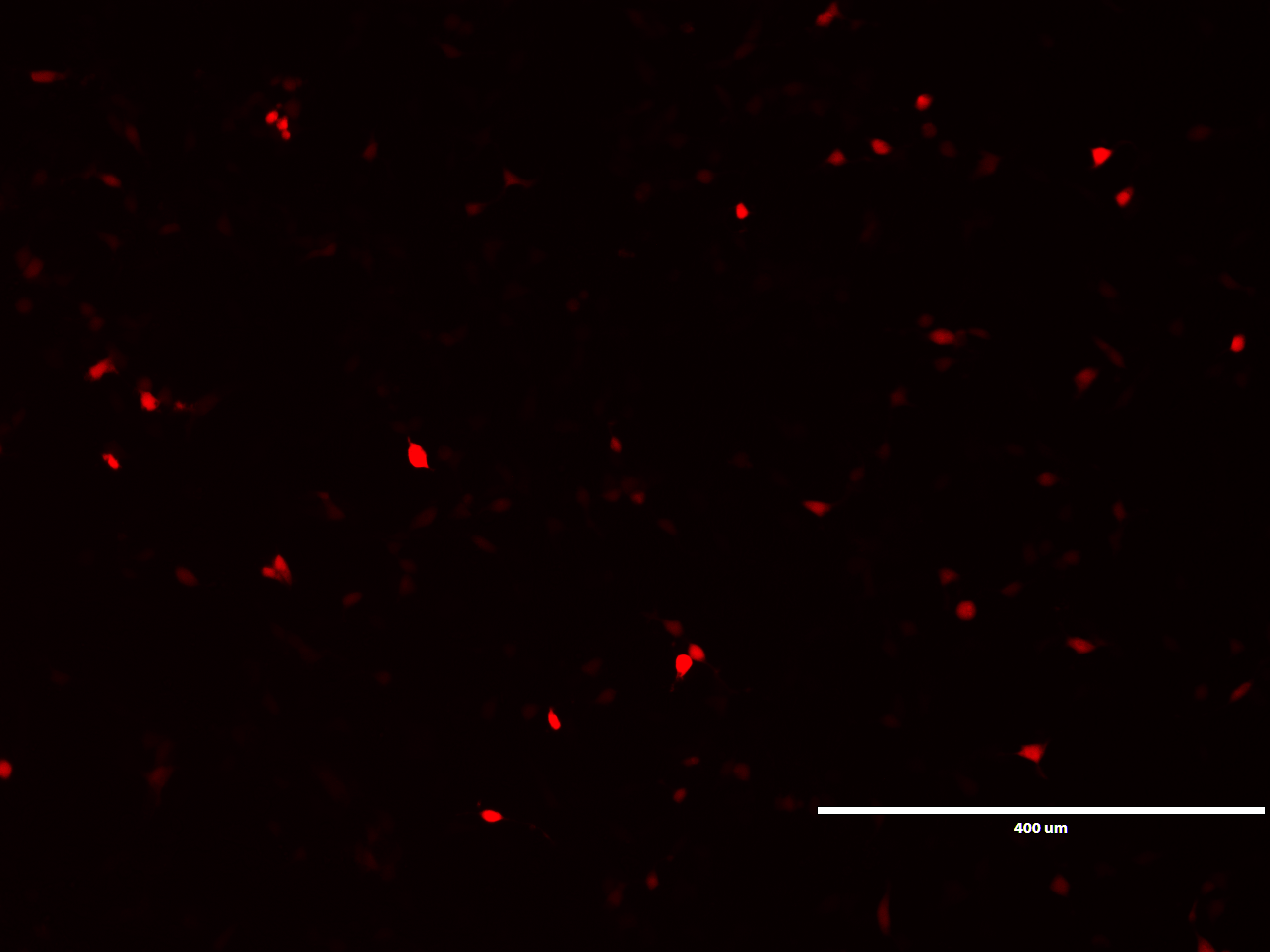

Supplement: Supplemental Information 5 [file peerj-10-13523-s005.zip › 2 the original files for any microscopy or flow cytometry data/microscopy data/Figure 1E/control.tif]
