# Supplementary material for: Muscone improves hypoxia/reoxygenation (H/R)-induced neuronal injury by blocking HMGB1/TLR4/NF-κB pathway via modulating microRNA-142
Source: PeerJ. 2022 Jul 15;10:e13523. doi: 10.7717/peerj.13523 (PMC9290999; doi:10.7717/peerj.13523)
Supplement: Supplemental Information 3 [file peerj-10-13523-s003.pdf]

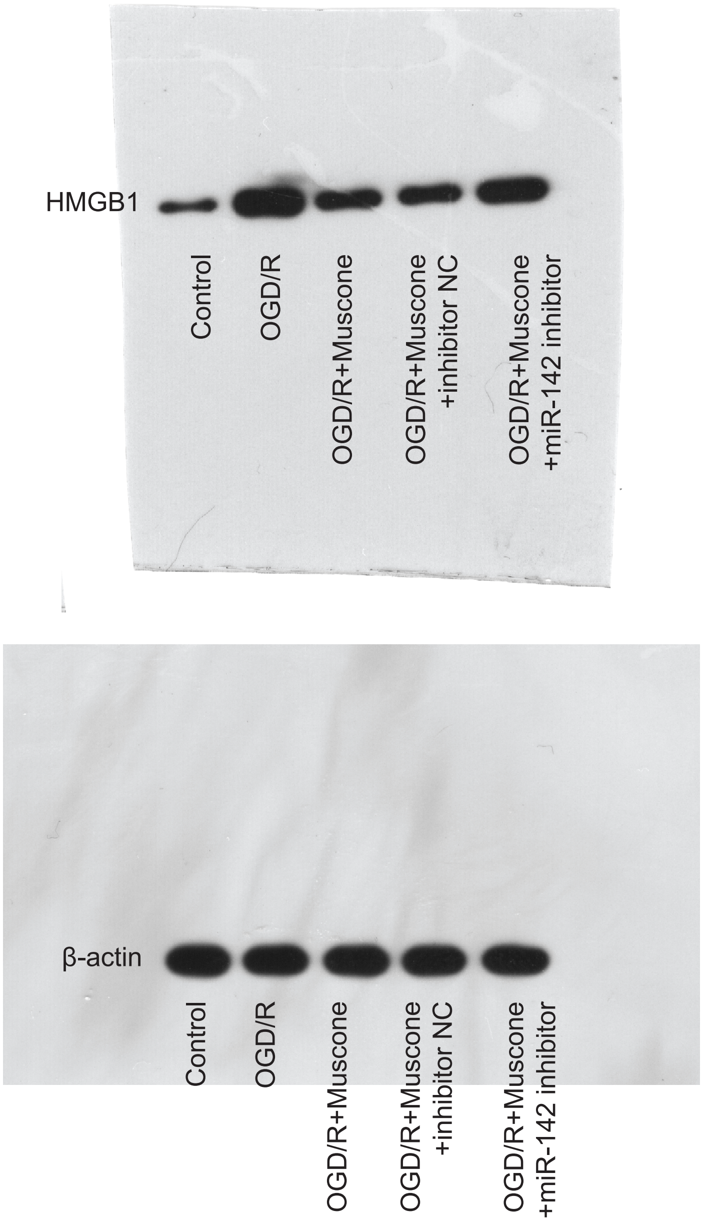
HMGB1
Control
OGD/R
OGD/R+Muscone
OGD/R+Muscone +inhibitor NC
OGD/R+Muscone +miR-142 inhibitor
β-actin
Control
OGD/R
OGD/R+Muscone
OGD/R+Muscone +inhibitor NC
OGD/R+Muscone +miR-142 inhibitor
